# Granulomatosis with polyangiitis

**DOI:** 10.36416/1806-3756/e20250091

**Published:** 2025-05-21

**Authors:** Roberta Wartchow Machado, Felipe Welter Langer, Dionatta Halle Flores Lisboa

**Affiliations:** 1. Departamento de Radiologia e Diagnóstico por Imagem, Hospital Universitário de Santa Maria, Santa Maria (RS) Brasil.; 2. Instituto de Radiologia - InRad - Hospital das Clínicas, Faculdade de Medicina da Universidade de São Paulo - HC-FMUSP - São Paulo (SP) Brasil.; 3. Universidade Federal de Santa Maria (RS) Brasil.

**Figure 1:**
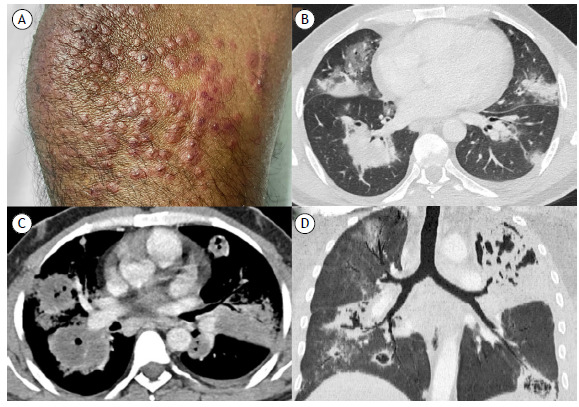
In A, clinical photograph showing palpable purpuric lesions in the lower extremities. In B, non-contrast-enhanced chest CT showing bilateral peribronchovascular consolidations with a surrounding halo of ground-glass opacity. In C, contrast-enhanced chest CT evidenced central cavitation and hypoenhancement within the pulmonary consolidations, suggestive of intralesional necrosis. In D, coronal non-contrast-enhanced chest CT with minimum intensity projection showing progression of the peribronchial lesions, most of which cavitated.

A 42-year-old male smoker presented with a two-month history of fever, hemoptysis, weight loss, and palpable purpura (Figure 1A). Chest CT revealed bilateral peribronchovascular consolidations with a halo of ground-glass opacity (Figure 1B). Follow-up imaging evidenced splenic infarction and progression of the pulmonary lesions, most developing central cavitation (Figures 1C-1D). Laboratory investigation disclosed anemia (10.3 g/dL), leukocytosis (26,000/mm³ with left shift), thrombocytosis (614,000/mm³), glomerulonephritis, complement consumption, elevated C-reactive protein (196 mg/L), and positive c-ANCA antibody; rheumatoid factor, antinuclear antibodies, and tuberculosis testing were negative. Segmentectomy with histopathological analysis confirmed pulmonary granulomatous vasculitis. These findings were compatible with granulomatosis with polyangiitis (GPA). A pulse of high-dose corticosteroid therapy was initiated, but the patient eventually passed away due to massive hemoptysis.

GPA, formerly known as Wegener’s granulomatosis, is a multisystem granulomatous vasculitis that affects small and medium-sized vessels of the upper respiratory tract, lungs, and kidneys.^1^ Diagnosis of GPA relies on clinical, laboratory, imaging, and histopathological findings. On imaging, identification of cavitating pulmonary lesions with peribronchovascular distribution and concomitant halo sign, as seen in this case, may help narrowing the differential diagnosis.^2^ ^,^ ^3^ Massive hemoptysis and diffuse alveolar hemorrhage are rare and life-threatening complications of GPA. Treatment usually involves systemic corticosteroids and immunosuppressive agents.
